# Testicular Regression Syndrome Presenting as a Nonpalpable Undescended Testis in Adulthood: A Case Report and Literature Review

**DOI:** 10.1155/criu/3994628

**Published:** 2026-04-01

**Authors:** Bartholomeo Nicholaus Ngowi, Alex Mremi, Ester Lekei, Orgeness Mbwambo, Jay Lodhia

**Affiliations:** ^1^ Department of Urology, Kilimanjaro Christian Medical Centre, Moshi, Tanzania, kcmc.ac.tz; ^2^ School of Medicine, KCMC University, Moshi, Tanzania, kcmuco.ac.tz; ^3^ Department of Pathology, Kilimanjaro Christian Medical Centre, Moshi, Tanzania, kcmc.ac.tz; ^4^ Department of General Surgery, Kilimanjaro Christian Medical Centre, Moshi, Tanzania, kcmc.ac.tz; ^5^ Department of Renal and Pancreatic Transplantation, Manchester University NHS Foundation Trust, Manchester, UK, mft.nhs.uk

**Keywords:** cryptorchidism, testicular regression syndrome, undescended testis, vanishing testis

## Abstract

Vanishing testis is also known as testicular regression syndrome, which is atrophy and disappearance of one testis during fetal life. Vanishing testis accounts for < 5% of undescended testis. Vanishing testis is a rare condition that must be remembered in the differential diagnosis of undescended testis. We report a case of vanishing testis in a young man of 29 years old who underwent inguinal exploration for nonpalpable undescended testis. There was no obvious testis at exploration and instead the vas deferens together with cord vessels ended blindly distally. The spermatic cord was excised and the patient fared well postoperatively.

## 1. Introduction

Vanishing testis is the disappearance of the one‐sided testis in fetal life. It is also called testicular regression syndrome (TRS) as it involves a spectrum of disorders depending on the stage of fetal development or early neonatal life at which the regression takes place [1]. Vanishing testis syndrome or TRS can be seen in less than 5% of cryptochordism cases [2]. The aetiology of TRS is regarded as the theory of an ischaemic event in early or late fetal life due to the dystrophic calcification, hemosiderin deposits and presence of giant cells [3]. Management is laparoscopic followed by inguinoscrotal exploration depending on the presence of the spermatic cord [2]. Herein, we present a case of a 29‐year‐old man who presented with the complaint of absent left testis where it was thought to have undescended testis; hence, surgical exploration was performed. However, at exploration, the vas deferens and other spermatic cord vessels were found to end blindly.

## 2. Case Presentation

A young man aged 29 years presented with a history of absence of left testis since birth. He denied any associated history of inguinal or scrotal swelling, or painful scrotum. His past medical history was uneventful for any surgery or chronic illness. On family and social history, he was a nonmarried, second‐year college student. Upon examination, he was healthy with normal male pubic hair distribution, circumcised normal‐sized penis and hypoplastic left hemiscrotum with nonpalpable testis on the ipsilateral hemiscrotum as well as along the left inguinal scrotal area. The right testis was palpable in the right hemiscrotum, firm in consistency and normal in size. Ultrasound of the abdomen and scrotum revealed an empty left hemiscrotum and left inguinal canal. There was no identifiable testicular tissue in the abdomen. However, the right hemiscrotum had a normal sized testis. Based on the above history, physical examination and ultrasound findings, a diagnosis of left undescended testis with a differential of varnishing testis was entertained. The patient was prepared for inguinal exploration due to the lack of laparoscopic equipment.

**Figure 1:**
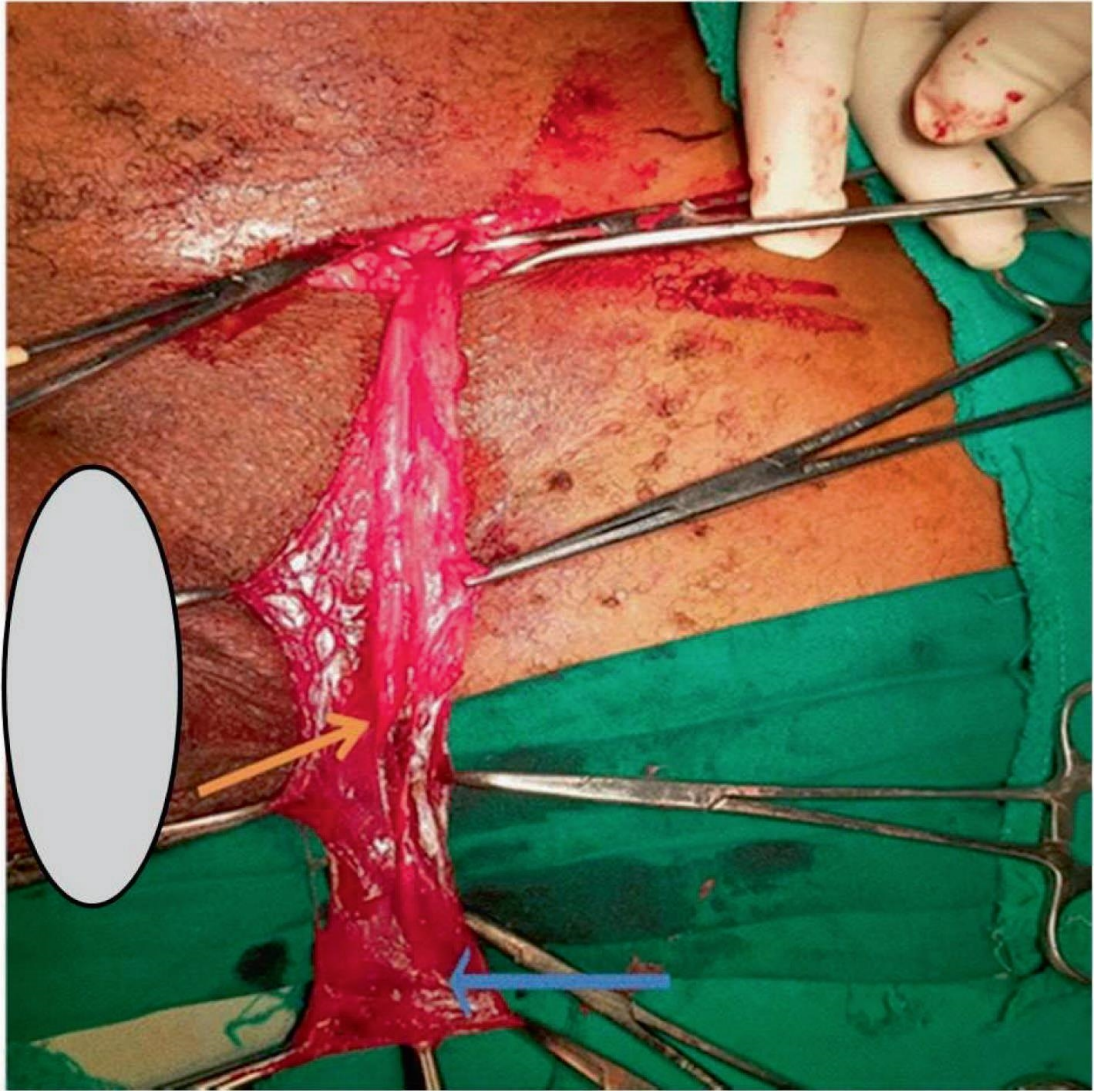
Photograph showing blind‐ending spermatic cord (blue arrow) and vas deferens (yellow arrow).

**Figure 2:**
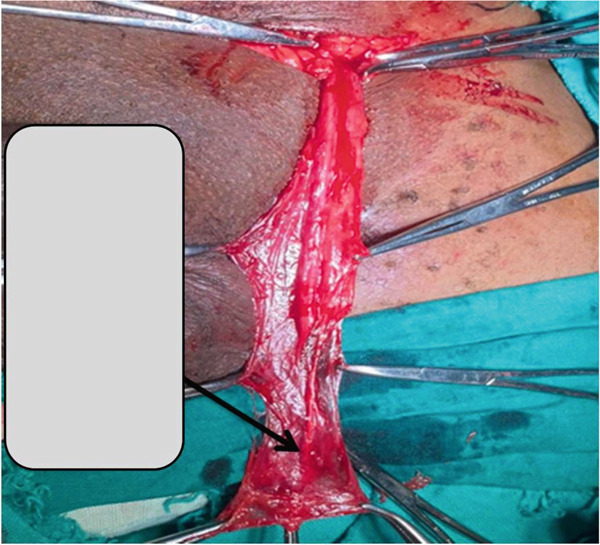
Photograph showing blind‐ending testicular vessel (black arrow).

Intraoperative, the spermatic cord was mobilised towards its scrotal end, where it was noted to end blindly (Figure 1). The vas deferens was tapered distally at its scrotal end and ended blindly. Similarly, the spermatic vessels ended blindly (atretic) at the distal end (scrotal end) of the spermatic cord where there was no obvious testis or nodule identified at the most distal end (scrotal part) of the vas deferens or spermatic vessels (Figure 2). Hence, a diagnosis of vanishing testis was confirmed and therefore, further exploration including abdominal exploration was not done. The spermatic cord was excised and the contralateral testis was fixed in the ispilateral hemiscrotum. The patient recovered well and on Day 2 postoperatively was discharged home. His 1‐year follow‐up at outpatient clinic was uneventful.

## 3. Discussion

Vanishing testis or TRS is a result of atrophy and ultimately disappearance of the normally formed testis [2].

The condition is clinically diagnosed by finding a blind‐ending spermatic cord at exploration for undescended testis/nonpalpable testis, a characteristic similar to this case [1, 2, 4]. Although cord structures are usually present, about 22.5% of cases present with complete absence of testis, vas deferens and epididymis; 15% have blind‐ending vas deferens; and 67.5% have blind‐ending vas deferens and blood vessels (atretic vessels), similar to this case [1, 2]. Presence of spermatic cord is the evidence that testis was present at some point in life, usually intrauterine [2]. Unlike in this case, a small nodule containing viable germ cell (GC) that occurs in 0%–16% of cases may be present at the end of spermatic cord [1, 2, 5].

Varnishing testis or TRS occurs in < 5% of undescended testis and 35%–60% of nonpalpable testis [2, 3, 5, 6]. Apart from vanishing testis, other causes of nonpalpable testis include testicular agenesis and intra‐abdominal testis [2]. The spectrum of external genitalia appearance ranges from normal male to phenotypic female, with a phenotypic male with micropenis in between the spectrum [2]. Most cases have a normal 46XY male with otherwise normal external genitalia [1–3]. Similarly, the index case had phenotypic normal male external genitalia except the absence of one testis in the scrotum.

The cause of vanishing testis is not well known but it is thought to be due to antenatal or perinatal torsion or endocrinopathy. Other causes include thrombus occluding the testicular vessel [1, 2, 5, 6]. Current studies support the vascular pathology and antenatal torsion rather than endocrinopathy as the cause of vanishing testis. These are supported by the presence of hemosiderin‐laden macrophage in the removed testicular nubbin that point towards haemorrhagic infarction and venous congestion [2, 6]. There has been a report of Y chromosome microdeletion. Most cases are sporadic but some have been reported to have a familial history [1].

Most cases of TRS are diagnosed early during routine newborn examinations or paediatric check‐ups in the first year of life. As late (adulthood) presentation is being rare [7], like with undescended testis, the delayed diagnosis of TRS may be attributed to misdiagnosis by a physician or a lack of insight by the parents [8]. Similarly, the index case was diagnosed late at the age of 29 years, most likely due to the same reasons as mentioned above.

In the English literature there is one case of TRS diagnosed in the 4th decade of life [7]. Late presentation, particularly for bilateral disease, is associated with complications of hypogonadism such as gynaecomastia, osteoporosis, erectile dysfunction and obesity. Infertility is another complication of long‐standing hypogonadism caused by bilateral TRS. Bilateral disease is associated with high mortality due to the effect of low testosterone on the cardiovascular system and glucose metabolism leading to diabetes mellitus, hypertension and hyperlipidaemia [7].

Like the index case, most cases of vanishing testis are unilateral, with the left side accounting for up to 68%. Bilateral TRS can occur but is rarely associated with normal external genitalia [1]. The predominance of the left side is probably due to its peculiar venous drainage that drains to the ipsilateral renal vein, and as a result, there may be a chance for retrograde flow of catecholamine from the left adrenal to the testis that might cause damage. Kinking of the left renal vein as a result of a highly mobile ipsilateral kidney may contribute to the vascular insult, as there is no venous anastomosis before 16 weeks of gestation [1, 4].

Diagnostic laparoscopic is the procedure of choice in cases of nonpalpable testis that include varnishing tests. However, in the absence of facilities for laparoscopy, open inguinal exploration is an alternative option as it was done for this case.

Controversy exists on the management of TRS, particularly on whether testicular remnants should be excised or not. The incidence of viable GCs and seminiferous tubules (SNTs) in TRS is 10% and 24%, respectively [9]. Presence of remnant tissue with GCs and SNTs is thought to pose a risk for testicular cancer formation in future [9]. However, the natural history of TRS remnant is largely unknown with intratubular GC neoplasm, a precursor to testicular cancer being rarely reported in TRS [9, 10]. Similarly, no case of invasive testicular cancer has been reported in association with TRS [10]. Currently, surgical excision of testicular remnant is the recommended option in TRS due to high incidence of viable GCs and SNTs which theoretically pose a risk for cancer formation [2, 3, 9, 10].

Although in the index case there was an obvious nodule distal to the spermatic cord (scrotal end), the cord was excised proximally at the level of the internal inguinal ring. Like any case with one testis, the contralateral testis is fixed to prevent testicular torsion, which is similar to what was done in our case [2, 3].

Vanishing testis is a rare occurrence and should be remembered as one of the differential diagnoses of undescended testis. We recommend excision of the spermatic cord together with the testicular nubbin in order to prevent the chance of malignant transformation. Further evidence‐based guidelines on evaluation and management of TRS are needed to reduce inconsistency.

## Funding

No funding was received for this manuscript.

## Consent

Written informed consent was obtained from the patient for publication for this case report; additionally, accompanying images have been censored to ensure that the patient cannot be identified. Ethical clearance was sought from the hospital administration. A copy of the consent is available on record.

## Conflicts of Interest

The authors declare no conflicts of interest.

## Data Availability

Data sharing is not applicable to this article as no datasets were generated or analysed during the current study.
